# Analysis of factors that influence the occurrence of otitis media with effusion in pediatric patients with adenoid hypertrophy

**DOI:** 10.3389/fped.2023.1098067

**Published:** 2023-02-22

**Authors:** Wenjing Chen, Guoping Yin, Yijing Chen, Lijun Wang, Yingying Wang, Chunmei Zhao, Wan Wang, Jingying Ye

**Affiliations:** ^1^ Department of Otolaryngology-Head and Neck Surgery, Beijing Tsinghua Changgung Hospital, School of Clinical Medicine, Tsinghua University, Beijing, China; ^2^ Department of Clinical Laboratory, Beijing Tsinghua Changgung Hospital, School of Clinical Medicine, Tsinghua University, Beijing, China

**Keywords:** adenoid hypertrophy, otitis media with effusion, influencing factor, obstructive sleep apnea, conditional pathogen

## Abstract

**Objective:**

Adenoid hypertrophy (AH) and otitis media with effusion (OME) are common pediatric otolaryngological diseases and often occur concurrently. The purpose of this study was to comprehensively analyze the factors that influence the occurrence of OME pediatric patients with AH.

**Methods:**

Patients younger than 12 years with AH, who were hospitalized for treatment at Beijing Tsinghua Changgung Hospital in Beijing, China, between March 2018 and February 2022 were enrolled. The patients were divided into an AH group and an AH + OME group based on the presence of OME. The authors collected the following clinical data for univariable analysis: sex; age; body mass index (BMI); comorbid nasal congestion/rhinorrhea, recurrent tonsillitis, or allergic rhinitis (AR); adenoid and tonsil grade; tonsillar hypertrophy; food/drug allergy; history of adenoidectomy and congenital diseases; breastfeeding status; preterm birth; exposure to environmental tobacco smoke (ETS); family history of adenotonsillectomy, otitis media, and AR; main data of polysomnography and oropharyngeal conditional pathogen culture data of some patients. Univariate analysis was performed as a basis for logistic regression analysis.

**Results:**

A total of 511 children (329 boys and 182 girls) were included, their mean age was 5.37 ± 2.10 years. Of them, 407 (79.6%) were in the AH group and 104 (20.4%) in the AH + OME group. Univariate analysis revealed statistically significant differences in age, BMI, adenoid grade, AR, breastfeeding status, and ETS exposure between the two groups. Multivariate stepwise logistic regression analysis showed that age, adenoid grade, AR, breastfeeding status, and ETS influenced the occurrence of OME in pediatric patients with AH. The risk of OME decreased with increasing age. High adenoid grade, ETS exposure, and comorbid AR were risk factors for OME in pediatric patients with AH, but breastfeeding was a protective factor. The final analytical results of the oropharyngeal conditional pathogen culture data showed that *Streptococcus pneumoniae* positivity was associated with OME in AH.

**Conclusion:**

The pathogenesis of AH with OME is complex. Young age, high adenoid grade, ETS exposure, non-breastfed status, comorbid AR, and the presence of *S. pneumoniae* in the oropharynx are risk factors for OME in pediatric patients with AH.

## 1. Introduction

Adenoid hypertrophy (AH) and otitis media with effusion (OME) are common pediatric otolaryngological diseases. Repeated stimulation by bacteria, viruses, and allergens causes pathological AH, resulting in clinical symptoms (1), such as nasal congestion, rhinorrhea, open-mouthed breathing, obstructive sleep apnea (OSA), snoring, and “adenoid face” caused by chronic airway obstruction, which is an important factor that can induce or worsen OME, eustachian tube dysfunction (ETD), and acute/chronic rhinosinusitis in children (2).

OME is a middle ear effusion without an acute middle ear infection. It is most common in children aged six months to four years old. Approximately 90% of preschoolers and 25% of schoolchildren have had OME (3). The prevalence of OME ranges from 1.3% to 31.3% (4) depending on diagnostic methods, race, and environmental factors. However, the pathogenesis and etiology of OME remain unclear. Viral infection, bacterial colonization, allergies, and immune factors can promote the occurrence and progression of OME; mechanical obstruction and ETD also play critical roles. Adenoids obstruct the posterior nostrils and affect ventilation and drainage in the nasal cavity and sinuses, leading to chronic sinusitis. Pathogenic microorganisms and secretions pass through the eustachian tube (ET) and oropharynx to cause ET mucosal inflammation, congestion, edema, and retrograde infection, thereby aggravating or inducing OME (5). AH is the main predisposing factor for OME that often accompanies it in children (6). However, not all pediatric patients with AH develop OME. It is vital to understand the influencing factors associated with OME incidence in pediatric patients with AH. Previous studies have found that atopic or allergic rhinitis (AR), frequent tonsillitis, daycare attendance, exposure to smoke, and multiple family members were major risk factors for OME in pediatric patients with AH (7). Further and more comprehensive analysis of the risk factors for OME in children with AH is needed to provide a reference for the prevention and treatment of such cases and a basis for future in-depth mechanistic studies. Thus, the aim of this study was to comprehensively analyze the factors that influence the occurrence of OME in pediatric patients with AH.

## 2. Materials and methods

### 2.1. Study population

In this retrospective study, patients with AH aged ≤12 years old who were hospitalized for treatment at Beijing Tsinghua Changgung Hospital in Beijing, China, between March 2018 and February 2022 were enrolled. The reasons for hospitalization were related clinical symptoms and manifestations of AH, mainly nasal congestion, rhinorrhea, open-mouthed breathing, OSA, snoring, and “adenoid face” caused by chronic airway obstruction. All patients were admitted to the hospital for an adenoidectomy. Some surgeries were combined with tonsillectomy. Two major indications for tonsillectomy and/or adenoidectomy include obstruction and recurrent infection (8). The age limit of 12 years was chosen because the upper age limit in OME guidelines is 12 years (9). Children with OME underwent intraoperative tympanocentesis or tympanostomy tube insertion. During the study period, a total of 518 patients were hospitalized due to the above reasons in an ENT outpatient department at our hospital. Seven were excluded (one case of undetermined neurological disease, one case of nephroblastoma, one case of abnormal coagulation function, one case of hereditary deafness with intellectual disability, one case of middle ear malformation, one case of immune disease, and one case of severe heart disease combined with slow growth). Finally, a total of 511 patients were included as study participants.

The inclusion criteria were as follows: age ≤ 12 years; presence of adenoid tissue obstruction of the posterior nostril (>50%); and presence of nasal congestion, snoring, mouth breathing, and other clinical symptoms. Patients who had acute upper respiratory tract infection in the last 2 weeks that was treated using antibiotics or immune modulators, and patients with cleft palate and other craniofacial deformities; intellectual disability; immunodeficiency; cardiovascular, lung, genetic, autoimmune, and neuromuscular diseases; or other severe underlying diseases were excluded from this study. The included patients were subdivided into an AH group and an AH + OME group based on the presence of OME, and into three groups by age: 0–4 years, 5–8 years, and 9–12 years.

Diagnosis of AH was based on: signs, symptoms, and the results of fiberoptic nasopharyngoscopy, and that of OME was based on: signs, symptoms, and the results of auxiliary examinations (pure tone audiometry/behavioral audiometry, tympanometry, and ear endoscopy), and the intraoperative confirmation of tympanic effusion.

The clinical data of the pediatric patients, including sex; age; body mass index (BMI); comorbid nasal congestion/rhinorrhea (mucoid or mucopurulent), recurrent tonsillitis, or AR; adenoid grade; tonsil grade; tonsillar hypertrophy; food/drug allergy; history of adenoidectomy; history of congenital diseases; breastfeeding status; preterm birth; exposure to environmental tobacco smoke (ETS); family history of adenotonsillectomy, otitis media and AR; and main polysomnography (PSG) data, were collected for univariate analysis. In addition, upper respiratory tract (oropharynx) conditional pathogen culture data were collected to analyze the relationship between the presence of conditional pathogens and the occurrence of OME in pediatric patients with OME.

The study design was approved by Beijing Tsinghua Changgung Hospital Ethics Committee (NO.: 22538-6-01, Nov. 8th, 2022). The minor(s)' legal guardian/next of kin consented to the collection of medical history, and examination and operation information.

### 2.2. Grouping criteria

The key points of diagnosis of OME were as follows: (1) ear symptoms and signs without acute middle ear infection; (2) hearing loss, self-hearing enhancement, or hearing changes with posture changes occuring; (3) a tympanogram showed a “B” or “C” curve; (4) pure tone/behavioral audiometry indicating that the affected ear had mild to moderate conductive hearing loss; and (5) patients who showed tympanic effusion during the ear endoscopy before the operation which was confirmed intraoperatively. Patients diagnosed with OME according to the above criteria were included in the AH + OME group. Pediatric patients without OME were included in the AH group.

### 2.3. Criteria for collection of clinical data

Adenoid grading was based on endoscopic findings of the percentage of the posterior nostril blocked by the adenoid. Grades II–IV indicate a 26%–50%, 51%–75%, and ≥76% obstruction, respectively (10). Tonsil grading was performed according to Friedman's criteria. The tonsil grades include grade 0 (patients who have had their tonsils removed), grade 1 (the tonsils are inside the tonsillar fossa), grade 2 (the tonsils extend beyond the tonsillar pillars), grade 3 (the tonsils extend beyond the tonsillar pillars but do not reach the midline), and grade 4 (the tonsils extend as far as the midline (11). Tonsil hypertrophy was defined as tonsil grade II or higher. Regarding breastfeeding status, included children were those who were breastfed for more than six months after birth. Children in close contact with at least one active smoker (one or more cigarettes per day by any family member living with them) were considered to have ETS exposure (12). AR was diagnosed if a child showed excessive sneezing and at least one of the following symptoms: ocular pruritus, nasal pruritus, oropharyngeal pruritus, or clear nasal discharge (13). Preterm birth was defined as a gestational age of fewer than 37 weeks at the time of delivery. Family history was defined as the medical history of first-degree relatives. PSG monitoring data were collected, including obstructive apnea index (OAI) and apnea hypopnea index (AHI). An OAI > 1 time/h or AHI > 5 times/h for every nighttime sleep was considered abnormal (14). The severity of OSA was categorized as follows: mild, 5 times/h < AHI ≤10 times/h or 1 time/h < OAI ≤ 5 times/h; moderate, 10 times/h < AHI ≤ 20 times/h or 5 times/h < AHI ≤ 10 times/h; severe, AHI > 20 times/h or OAI > 10 times/h. Bacterial culture sampling was performed after general anesthesia and before surgery. For the collection of the samples, 0.9% sodium chloride solution was used to flush the oropharynx, and sterile pharyngeal swabs were used to swab the oropharynx repeatedly. The samples were then sent for bacterial culture. These strains were identified using MALDI-TOF MS (Bruker Dalton GmbH, Leipzig, Germany). The samples were cultured on Columbia agar supplemented with 5% sheep blood and Chocolate agar plates and incubated at 37°C for 48 h.

### 2.4. Statistical analysis

SAS Analysis Software (version 9.4, SAS Institute Inc, Cary, NC, USA) was used for data processing. *P* < 0.05 was considered to be statistically significant. The study was mainly divided into two parts for statistical and data analysis. Firstly, the influencing factors of the clinical data of AH complicated by OME were analyzed. Then the relationship between different pathogenic bacteria and AH complicated by OME was further analyzed based on a limited number of cases. In the process, the relevant clinical data of the AH group and AH + OME group were first analyzed by univariate analysis. Measurement data meeting the normality test and homogeneity analysis of variance were compared between the two groups by T-test; otherwise, Wilcoxon non-parametric test was used. Pearson's Chi-square test was used for enumeration data meeting the condition of the test; otherwise, a correction test or Fisher's exact test was used. Then, variables with *P* < 0.1 from the univariate analysis results were included in the multivariate logistic stepwise regression to screen out related factors affecting the pediatric patients with AH complicated by OME. In the analysis of correlation with pathogenic bacteria, the relevant pathogenic bacteria were screened out by univariate analysis. Furthermore, variables with *P* < 0.05 were taken as covariables, and the relationship between pathogenic bacteria and OME in pediatric patients with AH was further analyzed by multivariate logistic regression.

## 3. Results

### 3.1. Analysis of clinical data

A total of 511 pediatric patients with AH were included in this study. Of the 511 patients, 329 were boys, and 182 were girls. The age distribution of the patients was 5.37 ± 2.10 years. Regarding the two patient groups, 407 (79.6%) patients were included in the AH group, whereas 104 (20.4%) were included in the AH + OME group. Thirteen of the patients were missing PSG data owing to a lack of parental consent or lack of cooperation by the pediatric patients. Of the 498 patients with PSG data, 397 (79.7%) were in the AH group, and 101 (20.3%) were in the AH + OME group. The upper respiratory tract conditional pathogen culture data of 220 pediatric patients were collected. Of these, 178 (80.9%) were in the AH group, and 42 (19.1%) were in the AH + OME group.

Univariate analysis of the clinical data of the patients (Table 1) showed that there were statistically significant differences in age, BMI, adenoid grade, AR, breastfeeding status, and ETS between the AH and AH + OME groups (*P* < 0.05). The age distribution of the patients in the AH group was 5 (4–7) years, whereas that of those in the AH + OME group was 4 (4–5) years, and the difference between the two groups was statistically significant (*P* < 0.0001). The incidence of OME was higher in younger children and significantly higher in the 0–4 years age group than in the older age groups (*P* = 0.0002). The BMI (kg/m^2^) of the pediatric patients in the AH group was higher than that of the patients in the AH + OME group (*P* = 0.0030). The incidence of OME among patients in the adenoid grade IV group was 26.86%, which was higher than that among patients in the grade III group (14.5%) (*P* = 0.0005). The incidence of OME among pediatric patients exposed to ETS was 36.72%, which was higher than that among patients who were not exposed to ETS (11.68%) (*P* < 0.0001). The incidence of OME among breastfed pediatric patients was 17.32%, which was lower than that among those not breastfed (29.23%) (*P* = 0.0036). The incidence of OME among patients with AR was 25.31%, which was higher than that among those without AR (15.93%) (*P* = 0.0085). There were no statistically significant differences in sex, tonsil grade, comorbid nasal congestion/rhinorrhea, history of adenoidectomy, history of congenital diseases, preterm birth, history of food/drug allergy, family history of otitis media, adenotonsillectomy and AR, PSG monitoring results: AHI, OAI, and diagnosis and severity of OSA between the AH and AH + OME groups (*P* > 0.05).

**Table 1:** Results of the univariate analysis of pediatric patients with adenoid hypertrophy with otitis media with effusion.

Variable	AH	AH + OME	*χ* ^2^	*P*-value
**Sex**			1.3379	0.2474
Male	257 (78.12)	72 (21.88)		
Female	150 (82.42)	32 (17.58)		
**Age (years)**	5 (4–7)	4 (4–5)	18.1938	<0.0001^b^
**Age group (years)**			16.7516	0.0002
0–4	164 (72.25)	63 (27.75)		
5–8	194 (83.62)	38 (16.38)		
9–12	49 (94.23)	3 (5.77)		
**BMI**	15.13 (13.96–16.9)	14.2 (13.66–16.21)	8.7825	0.0030^b^
**Nasal congestion /rhinorrhea**			0.2556	0.6132
No	67 (81.71)	15 (18.29)		
Yes	340 (79.25)	89 (20.75)		
**Allergic rhinitis**			6.9194	0.0085
No	227 (84.07)	43 (15.93)		
Yes	180 (74.69)	61 (25.31)		
**Recurrent tonsillitis**			0.0036	0.9523
No	283 (79.72)	72 (20.28)		
Yes	124 (79.49)	32 (20.51)		
**Adenoid grade**			12.0086	0.0005
III	230 (85.5)	39 (14.5)		
IV	177 (73.14)	65 (26.86)		
**Tonsil grade**			4.1044	0.3921
0	3 (60)	2 (40)		
1	31 (75.61)	10 (24.39)		
2	156 (77.23)	46 (22.77)		
3	164 (81.59)	37 (18.41)		
4	53 (85.48)	9 (14.52)		
**Tonsil hypertrophy**			2.7378	0.0980
No	190 (76.61)	58 (23.39)		
Yes	217 (82.51)	46 (17.49)		
**History of adenoidectomy**			0.0018	0.9666^a^
No	398 (79.76)	101 (20.24)		
Yes	9 (75)	3 (25)		
**History of congenital diseases**			0	1.0000^a^
No	399 (79.64)	102 (20.36)		
Yes	8 (80)	2 (20)		
**Preterm birth**			2.5011	0.1138
No	385 (80.38)	94 (19.62)		
Yes	22 (68.75)	10 (31.25)		
**Breastfeeding**			8.4788	0.0036
No	92 (70.77)	38 (29.23)		
Yes	315 (82.68)	66 (17.32)		
**History of food/drug allergy**			2.4518	0.1174
No	350 (80.83)	83 (19.17)		
Yes	57 (73.08)	21 (26.92)		
**Family history of otitis media**			0	1.0000^a^
No	389 (79.55)	100 (20.45)		
Yes	18 (81.82)	4 (18.18)		
**Family history of adenotonsillectomy**			0.2268	0.6339
No	381 (79.87)	96 (20.13)		
Yes	26 (76.47)	8 (23.53)		
**Family history of allergic rhinitis**			0.01274	0.9101
No	248 (79.49)	64 (20.51)		
Yes	159 (79.9)	40 (20.1)		
**Environmental tobacco smoke**			44.7721	<0.0001
No	295 (88.32)	39 (11.68)		
Yes	112 (63.28)	65 (36.72)		
**AHI**	3.8 (1.9–7.9)	4.6 (2.2–10.5)	1.1448	0.2846
**OAI**	0.1 (0–0.8)	0.2 (0–1.2)	1.5794	0.2088
**OSA**			2.2065	0.1374
No	233 (82.04)	51 (17.96)		
Yes	164 (76.64)	50 (23.36)		
**Severity of OSA**			4.0171	0.2596
No	233 (82.04)	51 (17.96)		
Mild	78 (78.79)	21 (21.21)		
Middle	53 (71.62)	21 (28.38)		
Severe	33 (80.49)	8 (19.51)		

Note: *t*-test was used for analysis of continuous variables, whereas Pearson's Chi-square test was used for analysis of categorical variables.

^a^ Continuity-adjusted Chi-square test was used.

^b^ Non-parametric test was used.

AH, adenoid hypertrophy; OME, otitis media with effusion; OAI, obstructive apnea index; AHI, apnea hypopnea index; OSA, obstructive sleep apnea.

The factors with a Pvalue <0.1 in the univariate analysis were included in the multivariate stepwise logistic regression analysis (Table 2). The results showed that age, adenoid grade, AR, breastfeeding status, and ETS exposure were important factors that influence the occurrence of OME in pediatric patients with AH. The results also showed that the risk of OME decreases with age. The patients in the 5–8 years [*P* = 0.0062, OR: 0.494 (0.299–0.819)] and 9–12 years [*P* = 0.0055, OR: 0.169 (0.048–0.592)] age groups had lower risks for OME than those in the 0–4 years group. The results also showed that a high adenoid grade was a risk factor for OME in pediatric patients with AH. The incidence of OME among patients in the adenoid grade IV group was 1.662 times than those in the grade III group [*P* = 0.0438, OR: 1.662 (1.014–2.723)]. ETS exposure was a risk factor for OME in pediatric patients with AH. Exposure to ETS increased the risk for OME by 4.839 times compared with non-exposure [*P* < 0.0001, OR: 4.839 (2.994–7.819)]. Children with AR were 1.906 times more likely to develop OME than those without AR [*P* = 0.008, OR: 1.906 (1.183–3.071)]. Breastfeeding was a protective factor against OME in pediatric patients with AH [*P* = 0.0126, OR: 0.523 (0.314–0.870)].

**Table 2:** Results of multivariate logistic regression analysis of pediatric patients with adenoid hypertrophy with otitis media with effusion.

Variable	Estimate	Standard Error	Wald χ^2^	*P*-value	OR	OR (95% CI)	
Age group (years)							
0–4	Ref.						
5–8	−0.7042	0.2573	7.4909	0.0062	0.494	0.299	0.819
9–12	−1.7805	0.6407	7.7234	0.0055	0.169	0.048	0.592
Adenoid grade							
III	Ref.						
IV	0.5079	0.2519	4.065	0.0438	1.662	1.014	2.723
Allergic rhinitis							
No	Ref.						
Yes	0.645	0.2433	7.0289	0.008	1.906	1.183	3.071
Breastfeeding							
No	Ref.						
Yes	−0.6482	0.2599	6.2215	0.0126	0.523	0.314	0.870
Environmental tobacco smoke							
No	Ref.						
Yes	1.5766	0.2449	41.4469	<.0001	4.839	2.994	7.819

### 3.2. Conditional pathogen culture analysis

The major pathogens identified in the upper respiratory tract (oropharynx) conditional pathogen culture analysis of the pediatric patients with AH included *Staphylococcus aureus*, *Streptococcus pneumoniae*, *Moraxella catarrhalis*, and *Haemophilus influenzae* (Table 3). Univariate analysis showed that the incidence of OME was 22.97% in the conditional pathogen-positive group, which was higher than that in the conditional pathogen-negative group (11.11%) (*P* = 0.0357). The incidence of OME was 34.88% in the *S. pneumoniae*-positive group, which was higher than that in the *S. pneumoniae*-negative group (15.25%) (*P* = 0.0033). The incidence of OME was 33.33% in the *H. influenzae*-positive group, which was higher than that in the *H. influenzae*-negative group (16.84%) (*P* = 0.0327). The difference between the two groups was statistically significant (*P* < 0.05).

**Table 3:** Relationship between conditional pathogen culture and patients with adenoid hypertrophy with otitis media with effusion.

Variable	AH	AH + OME	χ^2^	*P*-value
**Conditional pathogen**			4.4122	0.0357
No	64 (88.89)	8 (11.11)		
Yes	114 (77.03)	34 (22.97)		
**Streptococcus pneumoniae**			8.6300	0.0033
No	150 (84.75)	27 (15.25)		
Yes	28 (65.12)	15 (34.88)		
**Staphylococcus aureus**			3.6534	0.056
No	127 (77.91)	36 (22.09)		
Yes	51 (89.47)	6 (10.53)		
**Moraxella catarrhalis**			2.4219	0.1196
No	153 (82.7)	32 (17.3)		
Yes	25 (71.43)	10 (28.57)		
**Haemophilus influenzae**			4.5618	0.0327
No	158 (83.16)	32 (16.84)		
Yes	20 (66.67)	10 (33.33)		

The conditional pathogen culture analysis showed that age and adenoid grade were correlated with AH complicated by OME (*P* < 0.05) (Supplementary Table S1). Hence, after correcting for age and adenoid grade, the relationship between the presence of conditional pathogens and AH complicated by OME was analyzed (Table 4). Oropharyngeal *S. pneumoniae* positivity was found to be correlated with AH complicated by OME [*P* = 0.0161, OR: 2.647 (1.198–5.847)].

**Table 4:** Relationship between conditional pathogen culture and adenoid hypertrophy with otitis media with effusion (corrected for age and adenoid grade).

Variable	Estimate	Standard Error	Wald χ^2^	*P*-value	OR	OR (95% CI)	
Conditional pathogen							
No	Ref.						
Yes	0.5858	0.4428	1.7505	0.1858	1.796	0.754	4.279
Streptococcus pneumoniae							
No	Ref.						
Yes	0.9735	0.4043	5.797	0.0161	2.647	1.198	5.847
Hemophilus influenzae							
No	Ref.						
Yes	0.8834	0.4648	3.6123	0.0574	2.419	0.973	6.016

## 4. Discussion

OME is a major cause of hearing loss in children and can affect language and behavioral development (15). Epidemiological surveys have shown that more than 50% of children aged < 1 year and 60% of children aged <2 years have a history of OME (16). The high prevalence of OME among young patients is due to their incomplete structural and functional development of ET, which are affected by age-related ET factors, including length, angle, and closure capacity (17). In our study, age group analysis revealed that the proportion of pediatric patients with AH complicated by OME aged <4 years was higher than that of the other age groups. The results of the analysis also showed that the risk for OME decreased with increasing age. The difference between the two groups was statistically significant (*P* < 0.05). There is no consensus on the relationship between gender and OME. The disease is expected to be more common in boys as mastoid pneumatization is more rapid in girls and boys experience upper respiratory infection episodes more frequently. Some studies also showed that OME is more common in males (18). On the contrary, other studies showed no relationship between sex and the prevalence of OME (19–21). In our study, there was no statistical difference in sex distribution between the AH and AH + OME groups (*P* = 0.2475).

AH is the main cause of ETD, and OME is associated with ETD (10, 22). One study showed that 29.2% of children with adenoid enlargement had a co-morbidity of asymptomatic OME (23). The etiology of OME mainly includes anatomical, immune, microbiological, and environmental factors (6); however, its etiology is not completely clear. Hence, there is a need to examine the mechanisms and factors that influence AH complicated by OME. It is necessary to fully understand the risk factors related to OME incidence in pediatric patients with AH to better screen for and manage this disease. Our study fully collected various data from the etiology and mechanism. A comprehensive and in-depth analysis of these factors early can facilitate appropriate, timely intervention, thereby preventing disease progression. It provides a reference for the prevention and treatment of diseases. It also provides a basis and ideas for researching the deep mechanism and correlation of each influencing factor.

### 4.1. Mechanical obstruction

Hypertrophic adenoids, particularly tissues near the torus and pharyngeal opening of the ET, can directly compress and obstruct the ET, resulting in impaired middle ear drainage, negative middle ear pressure, mucosal exudation, and OME. Studies have shown that the degree of AH is significantly correlated with OME and that the greater the degree, the higher the incidence of OME. In addition, OME tends to persist, and conservative treatment tends to fail in cases of higher degrees of AH. Children with a higher grade of AH have a higher risk of OME persistence, leading to conservative treatment failure and requiring surgical intervention, but this study had a limited sample size of only 65 cases (24). The present study showed that the incidence of OME in the adenoid grade IV group was 26.86%, which was significantly higher than that in the grade III group (14.5%) (*P* = 0.0005). The incidence of OME in the adenoid grade IV group was 1.662 (1.014–2.723) times than that in the grade III group. A high adenoid grade is a risk factor for AH complicated by OME. The higher the adenoid grade, the greater the respiratory tract obstruction and the more severe the OSA in pediatric patients (25). However, PSG monitoring data analysis in this study revealed no significant correlation between the AHI, OAI, diagnosis and severity of OSA, and the incidence of OME in pediatric patients with AH (*P* > 0.05). This might be due to the population distribution in the present study and multiple potential factors. Further in-depth studies are required to clarify the relationship between these variables and the incidence of OME. Regardless, clinicians should be vigilant in managing pediatric patients with AH and OME who present with OSA. Timely screening and intervention should be performed in such cases.

### 4.2. Pathogenic microorganisms

OME may be a sequela of acute otitis media (AOM). OME tends to occur in cases of AH and ETD; however, the middle ear environment determines the occurrence of OME (26). The upper respiratory tract is an important region for the occurrence of otitis media. According to the pathogen reservoir theory, long-term retention of pathogen-carrying secretions in hypertrophic adenoid crypts can become a microorganism “reservoir”. Pathogens can reach the middle ear through the ET, causing disease. Bacteria detected in adenoid tests include normal bacteria and conditional pathogens in the nasopharynx. Conditional pathogens mainly include *S. pneumoniae*, *H. influenzae*, *S. aureus*, and *M. catarrhalis*. These pathogens can cause otitis media, nasal sinusitis, upper respiratory tract infections, pneumonia, and systemic diseases (26–30). Typical ear pathogens isolated from middle ear effusions, nasopharyngeal samples, and adenoid samples include *S. pneumoniae*, *H. influenzae*, and *M. catarrhalis*. In addition, multi-pathogen infections are often present in OME (31, 32). After viral infection of the upper respiratory tract occurs, the pathogens can ascend to the middle ear through the ET (33) and disrupt the respiratory tract flora. Viruses can cause bacteria to transform from commensal to pathogenic, disrupt the airway epithelial barrier, decrease mucus and cilia clearance, induce the host to provide nutrients to pathogens, and promote adhesion and virulence in ear pathogens (34). Oropharyngeal and nasopharyngeal pathogens in children tend to translocate to the middle ear owing to the structural characteristics of the ET and the middle ear negative pressure caused by AH (35). Some studies have compared the bacteriology of the adenoids and tonsils in children by culture, and found an overall similarity in the bacteria sampled from the surfaces of tonsils and adenoids of children (36, 37). Surface samples from the nasopharynx and oropharynx may easily be contaminated by saliva, tears, and other secretions. Other studies used 16S rRNA gene pyrosequencing. One study found that the microbiome differs between crypts of the adenoids and crypts of the tonsils, including the relative abundances of potential pathogens such as *H. influenzae*, *S. pneumoniae,* and *M. catarrhalis* (38). Another study has reported combined analyses of the adenoids and tonsils microbiome in pediatric. The microbiome was not significantly different at the phylum level between the adenoids and tonsils (39). In future studies, 16S rRNA gene pyrosequencing technology can be used to identify the differences in bacterial distribution. In addition, whole-genome sequencing should be conducted to analyze the specificity of bacterial capsules and virulence factors. In our study, conditional pathogen culture analysis of the oropharynx which represented the upper respiratory tract revealed that the conditional pathogens detected in the oropharynx of the pediatric patients mainly consisted of *S. aureus*, *S. pneumoniae*, *M. catarrhalis*, and *H. influenzae*. The results of the univariate analysis (Table 3) showed that the incidence of OME was higher in the conditional pathogen-positive group, *S. pneumoniae*-positive group, and *H. influenzae*-positive group than in the corresponding negative groups; the inter-group differences were statistically significant (*P* < 0.05). In addition, there were statistically significant differences in age and adenoid grade between the groups (*P* < 0.05) (Supplementary Table S1). After correcting for the effects of age and adenoid grade on the incidence of OME, the results showed that presence of *S. pneumoniae* in the oropharynx is a risk factor for the occurrence of OME in pediatric patients with AH. The incidence of OME in the *S. pneumoniae*-positive group was 2.647 times that in the *S. pneumoniae*-negative group [*P* = 0.0161, OR: 2.647 (1.198–5.847)] (Table 4). Future studies with larger sample sizes are needed for further analysis of these results.

### 4.3. Local immune dysregulation in adenoids and allergic reactions

The middle ear is an independent immune organ that is structurally connected to the ET and the upper respiratory tract. Based on the same airway principle, antigens that stimulate the nasal mucosa can also produce mucosal immune responses in the ET and the middle ear. Adenoids contain T and B lymphocytes in different developmental stages and are major secondary lymphoid organs that constitute Waldeyer's ring. They participate in innate and acquired immunity to resist upper respiratory tract infections in children. Local immune dysregulation decreases adenoid barrier function and increases the incidence of host OME. There are differences in adenoid lymphocyte subset distribution in pediatric patients with AH. Patients with AH + OME show higher T and B lymphocyte counts than those with AH only, which leads to increased local antigen-presenting dendritic cell count (40), increased capture of airway pathogens, decreased immune function, and increased host susceptibility. Immunoglobulins produced by B cells are an important defense mechanism against otitis media and other upper respiratory tract infections (41). The present study showed that AR increases the risk for OME in pediatric patients with AH. AR is an IgE-mediated type I hypersensitivity reaction that is characterized by increased vascular permeability, increased mast cells and related inflammatory cell secretion of histamine, leukotriene, and other inflammatory mediators, occurrence of mucosal edema and exudation, obstruction of the ET, and decreased cilia motility, resulting in OME. Allergies contribute to the occurrence and progression of AH and OME. The incidence of OME is significantly higher in pediatric patients with AR than in pediatric patients without AR (42, 43). However, the specific incidence of OME is determined by population characteristics and diagnostic criteria. A systematic review of the pathophysiology by which allergy increases the risk of otitis media showed that allergy is related to the occurrence and acute exacerbation of OME (44). Allergen stimulation causes local immune responses, elevated Th2 cytokine secretion, weakened upper respiratory tract mucosal barrier function, increased nasopharyngeal bacteria (45), and increased incidence of OME. IgE, IL-4, histamine, and eosinophil levels in middle ear effusions are increased in patients with OME. In patients with AR, IL-4, IL-5, IFN-*γ*, and histamine secretions in the nasal mucosa are increased (46). In a previous study of a mouse model of OME created by inducing middle ear infection, middle ear mucosal immune responses towards bacterial lipopolysaccharides were increased in the AR group (47).

Besides etiological factors, several immutable factors such as genetic factors and family history, and variable factors such as environment and lifestyle can affect the occurrence and progression of the disease (48). ETS exposure is also known as “secondhand smoking” or “passive smoking”. Animal studies have demonstrated that nicotine can stimulate the hypothalamus *α*3*β*4 nicotinic acetylcholine receptor to decrease appetite, energy intake, and body weight (49). Parental smoking is a common source of ETS exposure in children. Interventional measures should be employed to decrease ETS exposure in children and improve their health (50, 51). In the United States, 29.2% of adolescents are exposed to ETS (52).Thus, protocols for improving home-smoking behavior should be studied and implemented (53). ETS exposure and AR in children are associated with increased prevalence of eczema (54). Studies have shown that passive smoking and ETS exposure are environmental risk factors for OME in children (20, 55). Maternal smoking habits and the number of family members who are smokers are significantly correlated with the risk of otitis media in children aged < 4 years old (56). However, some studies did not show an association between ETS exposure and otitis media in children (19). Our study showed that ETS exposure is a risk factor for the occurrence of OME in pediatric patients with AH. The main harmful components of cigarettes, including nicotine, tar, carbon monoxide, and acrolein, can cause respiratory diseases (57) and destroy ET surfactants. Cilia toxins decrease ciliary beat frequency and cause the ET mucosa and cilia in children to be susceptible to damage. Thus, adult smoking can disrupt upper respiratory tract flora (58) and affect children.

Our study showed that the incidence of OME in breastfed pediatric patients was 17.32%, which was lower than that in the patients who were not (29.23%) (*P* = 0.0036). This indicates that breastfeeding is a protective factor against the occurrence of OME in pediatric patients with AH (Table 2). Breast milk not only contains antibacterial substances, but can also promote the development of healthy flora and is negatively correlated with respiratory tract infections (59, 60). In addition, breast milk can optimize immune function and decrease the risk of otitis media and respiratory tract infections in infants and toddlers (61–63). A Lancet paper analyzed the potential mechanisms underlying the effects of breastfeeding in terms of immunology, epigenetics, microbiomics, and stem cell research. Increased breastfeeding can decrease rate of mortality in children and prevent infectious diseases. In addition, the protective effects of breastfeeding against otitis media in children can extend up to age 2 and older (64). In addition to providing environmental pathogen-specific IgA, breast milk can develop non-specific defenses against bacterial pathogens. Besides preventing upper respiratory tract infections, maternal antibodies can also act on middle ear pathogens and interfere with bacterial adhesion to the nasopharyngeal epithelium to prevent acute otitis media (65). Antigen stimulation can result in the production of IgG antibodies against non-typeable *H. influenzae* (66) and regulate an infant's humoral immune responses towards common OME pathogens. Milk bottle suction and the swallowing pressure gradient can cause ETD and increase susceptibility to otitis media (67).

In summary, the pathogenesis of AH with OME is complex and is influenced by many factors. Younger age and a high adenoid grade are major risk factors for the occurrence of OME in pediatric patients with AH. ETS exposure, a non-breastfed status, AR, and presence of conditional pathogens (mainly *S. pneumoniae*) in the upper respiratory tract also influence the pathogenesis of AH complicated by OME. This study provides a foundation and basis for future etiological mechanism studies and treatments. It is necessary to study deep mechanisms and correlations in the future, such as the establishment of animal models related to the incidence of OME influencing factors and whole-genome sequencing for the analysis of the specificity of bacterial capsules and virulence factors.

The presented study still had some main limitations. The sample sizes were limited, especially the cases of conditioned pathogen culture. In addition, the study time range included the global COVID-19 pandemic, which involved Beijing's strict implementation of the dynamic zero-COVID policy during this period. The prevalence of the novel coronavirus did not affect the incidence and progression of the disease in the study population. However, the overall number of patients hospitalized for AH and related diseases decreased compared to before the epidemic. In particular, there were very few patients from other cities, which seemed to have a potentially unavoidable effect on the distribution of the study population.

## Data Availability

The raw data supporting the conclusions of this article will be made available by the authors, without undue reservation.
