# Supplementary material for: Analysis of factors that influence the occurrence of otitis media with effusion in pediatric patients with adenoid hypertrophy
Source: Front Pediatr. 2023 Feb 22;11:1098067. doi: 10.3389/fped.2023.1098067 (PMC9992982; doi:10.3389/fped.2023.1098067)
Supplement: Supplementary file 1 [file Table1.pdf]

Supplementary Material

Supplementary Table 1. Results of univariate analysis of the conditional pathogen culture data of 220 pediatric patients with adenoid hypertrophy with otitis media with effusion

Variable	AH	AH+OME	χ2	P
**Sex**			0.0002	0.9898
Male	110 (80.88)	26 (19.12)		
Female	68 (80.95)	16 (19.05)		
**Age (years)**	5 (4–7)	4 (4–5)	18.1938	<0.0001[b]
**Age group (years)**			15.0453	0.0005
0–4	77 (70.64)	32 (29.36)		
5–8	81 (90)	9 (10)		
9–12	20 (95.24)	1 (4.76)		
**BMI**	15.115 (13.94–16.53)	14.04 (13.62–16.26)	3.7863	0.0517[b]
**Nasal obstruction/rhinorrhea**			0.1638	0.6857
No	30 (83.33)	6 (16.67)		
Yes	148 (80.43)	36 (19.57)		
**Allergic rhinitis**			0.3213	0.5708
No	106 (82.17)	23 (17.83)		
Yes	72 (79.12)	19 (20.88)		
**Recurrent tonsillitis**			0.0896	0.7647

No	136 (80.47)	33 (19.53)		
Yes	42 (82.35)	9 (17.65)		
**Adenoid grade**			10.2093	0.0014
III	91 (90.1)	10 (9.9)		
IV	87 (73.11)	32 (26.89)		
**Degree of tonsils**			——	0.7721
1	13 (92.86)	1 (7.14)		
2	88 (79.28)	23 (20.72)		
3	62 (80.52)	15 (19.48)		
4	15 (83.33)	3 (16.67)		
**History of adenoidectomy**			0	1.0000[a]
No	177 (80.82)	42 (19.18)		
Yes	1 (100)	0 (0)		
**History of congenital diseases**			——	0.3461[c]
No	177 (81.19)	41 (18.81)		
Yes	1 (50)	1 (50)		
**Preterm birth**			0.6792	0.4099[a]
No	165 (80.1)	41 (19.9)		
Yes	13 (92.86)	1 (7.14)		

**Breast feeding**			0.1171	0.7322
No	34 (79.07)	9 (20.93)		
Yes	144 (81.36)	33 (18.64)		
**History of food/drug allergy**			0.0016	0.9678
No	153 (80.95)	36 (19.05)		
Yes	25 (80.65)	6 (19.35)		
**Family history of otitis media**			0	1.0000[a]
No	164 (80.79)	39 (19.21)		
Yes	14 (82.35)	3 (17.65)		
**Family history of adenotonsillectomy**			0	1.0000[a]
No	162 (81)	38 (19)		
Yes	16 (80)	4 (20)		
**Family history of allergic rhinitis**			2.6149	0.1059
No	103 (77.44)	30 (22.56)		
Yes	75 (86.21)	12 (13.79)		
**Environmental tobacco smoke**			0.0066	0.9353
No	92 (80.7)	22 (19.3)		
Yes	86 (81.13)	20 (18.87)		

**Conditional pathogen**			4.4122	0.0357
No	64 (88.89)	8 (11.11)		
Yes	114 (77.03)	34 (22.97)		
Streptococcus pneumoniae			8.6300	0.0033
No	150 (84.75)	27 (15.25)		
Yes	28 (65.12)	15 (34.88)		
Staphylococcus aureus			3.6534	0.056
No	127 (77.91)	36 (22.09)		
Yes	51(89.47)	6 (10.53)		
Moraxella catarrhalis			2.4219	0.1196
No	153 (82.7)	32 (17.3)		
Yes	25 (71.43)	10 (28.57)		
Haemophilus influenzae			4.5618	0.0327
No	158 (83.16)	32 (16.84)		
Yes	20 (66.67)	10 (33.33)		

Note: t-test was used for analysis of continuous variables, whereas Pearson's Chi-square test was used for analysis of categorical variables.
[a]Continuity-adjusted chi-square test was used for analysis.
[b]Non-parametric test was used for analysis.
[c]Fisher's exact test was used for analysis.
Abbreviations: AH, adenoid hypertrophy; OME, otitis media with effusion; BMI, body mass index
